# Supplementary material for: Genome-wide Interaction Study Implicates VGLL2 and Alcohol Exposure and PRL and Smoking in Orofacial Cleft Risk
Source: Front Cell Dev Biol. 2022 Feb 10;10:621261. doi: 10.3389/fcell.2022.621261 (PMC8866867; doi:10.3389/fcell.2022.621261)
Supplement: Supplementary file 3 [file DataSheet1.docx]

**Supplemental Note**

*Simulation*

The two-stage approach for identifying GE effects used in these analyses is defined by:

1. Fit full interaction model $logit\left( p \right)=\beta_{0} +\beta_{1} * G +\beta_{2} * E +\beta_{3} * G * E$
2. Screen variants using the joint G-GE (2-df) test for $H_{0}: \left[ \begin{matrix} \beta_{1} \\ \beta_{3} \end{matrix} \right]= 0$ keeping those with $p_{2df}<\alpha_{1}$
3. Of remaining variants, use the GE test for $H_{0}:\beta_{3}=0$ using a significance threshold of $p_{GE}<\alpha_{2}$

The choice of $\alpha_{2}$ must be such that the dependence between the joint G-GE and GE tests is accounted for. To estimate the value of $\alpha_{2}$ given a value of $\alpha_{1}$, we performed a simulation study under the null hypothesis of no association. We simulated 500 participants with a binary outcome ($Y$), binary environmental exposure ($E$), and an additively coded genotype ($G$) such that $P(Y = 1) = 0.50$, $P(E = 1) = 0.50$, and the minor allele frequency (MAF) of 40% under Hardy-Weinberg Equilibrium (i.e., $P(G = 0) = 0.36, P(G = 1) = 0.48, P(G = 2) = 0.16$).

Using 10,000 simulations using the above parameters, we generated null distributions of p-values for the joint G-GE (2-df) test and the GE test. As expected, both the joint G-GE (2-df) test and the GE test alone achieve proper type-1 error control under the null hypothesis, given the choice of $\alpha$ (Figure S1); for example, 4.9% (490/10,000) simulations contained $p_{2df}<0.05$ and 5.3% (530/10,000) simulations contained $p_{GE}<0.05$.

**Figure S1. Quantile-quantile plots of observed p-values for the (A) joint G-GE and (B) GE tests from simulation versus those expected under a uniform(0,1) distribution.**


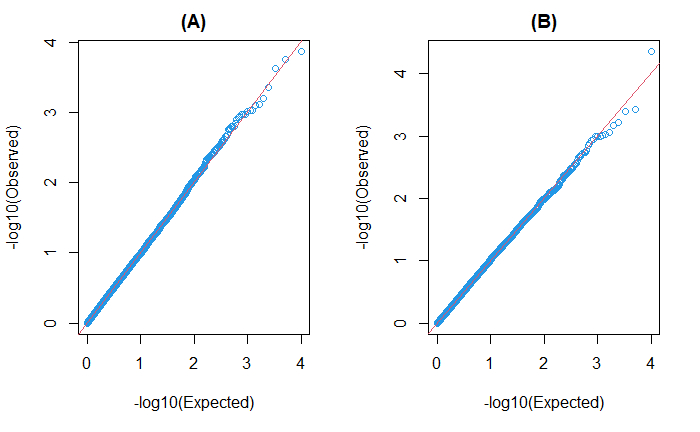


To estimate $\alpha_{2}$ for the second stage, we assumed a first-stage $\alpha_{1}$ of 0.05 and found the 5^th^ percentile of the simulated $p_{GE}$ for the filtered results with $p_{2df}<0.05$ . This corresponded to an empirical $\alpha_{2}$ of 0.00275, i.e., 5.1% (25/490) of the simulations that had $p_{2df}<0.05$ contained $p_{GE}<0.00275$.

Thus, to identify statistically significant associations, we used a first-stage screening procedure of $p_{2df}<0.05$ and a second-stage p-value threshold of $p_{GE}<0.00275$ to create a procedure with an overall type-1 error rate of 0.05.
